# ‘Manage and mitigate punitive regulatory measures, enhance the corporate image, influence public policy’: industry efforts to shape understanding of tobacco-attributable deforestation

**DOI:** 10.1186/s12992-016-0192-6

**Published:** 2016-09-20

**Authors:** Kelley Lee, Natalia Carrillo Botero, Thomas Novotny

**Affiliations:** 1 Faculty of Health Sciences, Simon Fraser University, Blusson Hall, 8888 University Drive, Burnaby, BC V5A 1S6 Canada; 2 Division of Epidemiology and Biostatistics, San Diego State University, San Diego, CA USA

**Keywords:** Tobacco industry, Leaf farming, Deforestation, Policy influence, Corporate social responsibility

## Abstract

**Background:**

Deforestation due to tobacco farming began to raise concerns in the mid 1970s. Over the next 40 years, tobacco growing increased significantly and shifted markedly to low- and middle-income countries. The percentage of deforestation caused by tobacco farming reached 4 % globally by the early 2000s, although substantially higher in countries such as China (18 %), Zimbabwe (20 %), Malawi (26 %) and Bangladesh (>30 %). Transnational tobacco companies (TTCs) have argued that tobacco-attributable deforestation is not a serious problem, and that the industry has addressed the issue through corporate social responsibility (CSR) initiatives.

**Methods:**

After reviewing the existing scholarly literature on tobacco and deforestation, we analysed industry sources of public information to understand how the industry framed deforestation, its key causes, and policy responses. To analyse industry strategies between the 1970s and early 2000s to shape understanding of deforestation caused by tobacco farming and curing, the Truth Tobacco Documents Library was systematically searched. The above sources were compiled and triangulated, thematically and chronologically, to derive a narrative of how the industry has framed the problem of, and solutions to, tobacco-attributable deforestation.

**Results:**

The industry sought to undermine responses to tobacco-attributable deforestation by emphasising the economic benefits of production in LMICs, blaming alternative causes, and claiming successful forestation efforts. To support these tactics, the industry lobbied at the national and international levels, commissioned research, and colluded through front groups. There was a lack of effective action to address tobacco-attributable deforestation, and indeed an escalation of the problem, during this period.

**Conclusions:**

The findings suggest the need for independent data on the varied environmental impacts of the tobacco industry, awareness of how the industry seeks to work with environmental researchers and groups to further its interests, and increased scrutiny of tobacco industry efforts to influence environmental policy.

## Introduction

Deforestation is the process whereby natural forests are cleared through logging and/or burning, either to use the timber or the area for alternative uses [1]. Around 1.5 billion hectares of forests, mainly tropical forests, have been lost since the 1970s [2], causing up to 30 % of greenhouse gas emissions annually [3]. Tobacco farming and curing constitute a “proximate cause” of deforestation due to forest removal for agricultural land, soil nutrient depletion and wood fuel usage [4]. Concerns about tobacco-attributable deforestation began to be raised in the mid 1970s [5], spurred by a 128 % increase in production in low- and middle-income countries (LMICs) [6], and 40 % increase globally from 1971 to 1997 [7]. Geist [8] (p. 18) estimated that, during the mid 1980s, “Virginia (flue-cured) tobacco consume[d] between 82.5 and 175 million cubic metres of roundwood harvested worldwide each year for curing…the equivalent of 1.2–2.5 million hectares of open forests or woodlands removed annually”. However, the problem continued unabated. Available data suggests tobacco caused 4 % of deforestation globally [9], but was a significant cause of deforestation in selected LMICs such as China (18 %) [8], Zimbabwe (20 %) [10], Malawi (26 %) [11] and Bangladesh (>30 %) [12].

The tobacco industry has claimed for decades that concerns about tobacco-attributable deforestation are based on misperception and myths, and it has been a minor cause of the problem. The industry has also been involved in mitigating activities in LMICs, notably tree planting (forestation), development of more efficient curing methods, and alternative fuels. By the late 1990s, these efforts formed a core part of the corporate social responsibility (CSR) initiatives of several transnational tobacco companies (TTCs). Today, TTCs claim that tobacco-attributable deforestation is being effectively addressed. In 2015, for instance, British American Tobacco (BAT) reported the planting of over 170 million trees, and 94.8 % of wood used for curing by its contracted farmers not coming from natural forests [13]. Philip Morris International (PMI) described its replenishing of forests and improving of wood use efficiency [14], while Imperial Tobacco [15] argues that its partnerships are “helping farmers to become self-sufficient in wood”.

This paper argues that there is a need to locate industry claims within a fuller account of the deforestation problem, and how the tobacco industry has sought to shape environmental research and policy debates from the 1970s to early 2000s. While tactics used by the tobacco industry to influence health policy are well-documented [16], there has been limited analysis of its efforts to influence environmental policy. Bringing together how the industry has publicly framed deforestation, with internal industry documents, this paper describes the tactics used by the industry to shape the definition of, and policy responses to, the problem of tobacco-attributable deforestation. The findings raise questions about the tobacco industry’s full impacts on the environment, and its efforts to engage with environmental researchers and groups through its CSR activities.

## Background

Large-scale commercial tobacco farming dates from the seventeenth century when expanding European demand led to the establishment of plantations in the Americas worked by African slaves. Over the next four centuries booming demand, and the ability of the plant to grow in most environments, saw tobacco farming spread worldwide [17]. Tobacco production comprises three sectors: growing and curing, leaf processing and manufacturing. The first is the responsibility of farmers who must deal with the “preparation of farms, nursery establishment, planting, farm/crop management, harvesting, curing, sorting and leaf grading, and transportation from their homes to leaf buying centres” [18] (p. 5). Leaf processing is dominated by large transnational companies, led by Universal Leaf and Alliance One, and their subsidiaries. Some large manufacturers also operate in tobacco growing countries and purchase leaf directly from farmers. In both cases, it is common that loans and other inputs (e.g. seeds, fertilizers) are provided to farmers under contract by the leaf purchaser up front, who then purchases the ready-cured leaf at a pre agreed price.

Leaf farming has shifted to LMICs since the 1960s, for both domestic consumption and a cash crop, reaching 90 % of world production by 2008 [19]. In 2015 tobacco was grown on 4.3 million hectares in 124 countries, producing 7.5 million tonnes of leaf [20].

Given the above business model, hundreds of thousands of small farmers in LMICs are now dependent on growing and curing tobacco for a global oligopoly of processors and manufacturers. In Malawi following independence in 1964, for example, development policies favoured tobacco-growing estates, many controlled by political elites [21]. Production more than quadrupled from 1961 to 1979 [22] (Table 5.1). By 2004, tobacco accounted for 70 % of exports [23] (p.274), 13 % of gross domestic product (GDP), and 23 % of total tax base [24]. In 2009, tobacco employed 40 % of Malawi’s total workforce [25]. Similarly, production more than doubled in Zimbabwe from 1980 to 1998 [22] (Chapter 7), with small-scale tobacco farms producing 50 % of agricultural exports, 30 % of total exports and 10 % of gross domestic product by 2003. In the Urambo district of Tanzania, 90 % of households surveyed were farmers who depended on crop production as the sole source of income, with 75 % of these farmers regular tobacco growers [26].

Importantly, the control of leaf supply through the contract system has led to “[s]teered overproduction…to keep tobacco leaf prices low at the farm gate to the disadvantage of the tobacco farmers” [18] (p.7). Global leaf production increased between 1970 and 1998 by almost 50 % [18] (p.7), while the real price of flue-cured leaf per tonne fell by 37 % from 1985 to 2000 [23] (p.274). As Otañez et al. write, the structure of leaf production “restrict[s] competition, depress tobacco prices for Malawi’s farmers and contribute to poverty in Malawi, while keeping the country dependent on tobacco growing” [25] (p.261). Alongside the impoverishment of smallholder farmers, and dependence of leaf exporting countries on an increasingly globalized tobacco industry, overproduction of tobacco leaf has created environmental impacts in the form of large-scale forest removal, soil nutrient depletion, heavy use of pesticides and chemicals, and intensive water and wood fuel usage [4]. It is in this context that the tobacco industry’s efforts to shape understanding of deforestation, caused by tobacco farming and curing since the 1970s, and the potential solutions can be located.

## Methods

We began by reviewing the existing scholarly literature to understand how environmental scientists see the problem of tobacco-attributable deforestation. We searched the Environment Complete database using the keywords “tobacco” and “*forestation”. We then examined company websites, reports, and industry publications (e.g. *Tobacco Journal International*, *Tobacco Reporter*) to identify how the industry has publicly framed the deforestation issue. Of particular interest were industry views put forth on the impacts of the problem, key causes, and industry responses. Materials reviewed include annual reports, company statements, public commentary, and news releases. To identify and analyse industry strategies to shape understanding of deforestation caused by tobacco farming and curing, from the 1970s when concerns began to be raised, the Truth Tobacco Documents Library was systematically searched between December 2014 and July 2015 using such keywords as “*forestation”, “wood”, “fuel”, combined together using Boolean terms. A snowball method was used to identify additional keywords generated from initial searches such as names of individuals, organizations, projects and countries. The timeframe for this analysis is limited by currently available documents which largely date to the early 2000s. The above sources were compiled and triangulated, thematically and chronologically, to derive a narrative of the industry strategy and activities related to deforestation.

## Results

### “Our significant common interests”: claiming economic solidarity with tobacco farmers in the developing world

Tobacco-attributable deforestation first raised international concern at the 1979 World Conference on Smoking and Health [27]. Attention was drawn by World Health Organization [WHO] [28] to a study [29] reporting that woodfuel curing requires one tree per 300 cigarettes. To counter these concerns, the industry initiated a “pro-active strategy” against “WHO’s propagandist views” focusing on “common interests” between the industry and farmers [30]. This initially focused on the industry-funded front group, International Committee on Tobacco Issues (ICOSI), later renamed the International Tobacco Information Centre (INFOTAB), which commissioned the Economic Intelligence Unit (EIU) to undertake “in-depth studies…in Brazil, Costa Rica, Nigeria, India and Malaysia” [31] (p. 1). The resultant report, *Leaf tobacco - its contribution to the economic and social development of the Third World*, then underpinned a “plan of action”, overseen by a newly created ICOSI Developing Countries Group, to alert governments and “provoke reaction in the Third World” [32]. TTCs were instructed by ICOSI to ensure “as many copies as possible reach people of influence in government departments and the media” [33] (p. 1). BAT informed its Territorial Coordinators, for example, that the “TAC [Tobacco Advisory Council, the British tobacco industry trade and lobbying group], [BAT] Public Affairs Department and other UK manufacturers will be giving out a number of copies to influential people in Government and the media here in the UK” [31] (p. 1). BAT described the report as providing “information not only to demonstrate the economic and social value of tobacco, but…to refute ill-considered WHO policies damaging to the interests of developing countries” [30]. ICOSI also considered ways “of ensuring that the World Bank, IMF [International Monetary Fund] and Western Countries’ Overseas Development Ministers are fully aware of” the report [32] (p. 15).

At the same time, industry involvement in the research was intentionally played down: “In accordance with ICOSI policy, a low profile approach would be necessary, probably through third parties” [32] (p. 15). ICOSI Secretary General Julian Doyle described how questions regarding independence were to be handled:

the industry provided support in terms of local liaison facilities and certain financial assistance. “The industry” in that context was the member companies of ICOSI and, if questioned on their role, they will be stating that they commissioned the study. I believe that this study, undertaken by a research organization second to none in international reputation for objective high quality work, will provide valid evidence of the industry’s very real contribution to economic and social development in the Third World. [34] (pp. 1–2)

The public relations firm Hill and Knowlton also recommended that the “independence” of the report be emphasized: “The EIU Study offers the best - yet independent evidence that tobacco growing is of great benefit and financial importance to underdeveloped regions of the world” [35].

Building on the 1980 report, and ahead of the 1983 World Conference on Smoking and Health, INFOTAB commissioned *The Role of Tobacco and Comparable Cash Crops in Rural Development in Selected Low Income Countries* by J.P. McInerney at the Centre for Agricultural Strategy, University of Reading. The purpose of the review was “to establish the economic contribution of….tobacco and its alternatives in the farming systems of small producers with the implications for income, employment and other parameters of rural development” [36]. Head of BAT Public Affairs Department Robert Ely wrote to colleagues that “Dr Burley and Mr S Rossides of EIU were mentioned as contacts for the Reading team should they wish to get in touch. A full copy of the EIU 1979 Paper would be sent to the Reading team together with any other material including economic impact studies which might be available” [37]. PM President Hamish Maxwell described the Reading study as a way of extending the industry’s “circle of allies” to “new constituencies”, advising that “[w]e must continue to build up in the professional literature a reservoir of credible articles like his which the industry can cite in defense of tobacco as a major contributor to cash-crop economies” [38] (pp. 12–15).

The industry began to lobby officials at the Food and Agriculture Organization (FAO) soon after, using the EIU and Reading studies, facilitated by former UN official turned industry consultant Niki Hauser. According to Mercer, the industry “knew that the World Health Organisation exerted…a significant pressure on FAO to support its anti-smoking campaigns” [39] (p.3). In a draft speech to ministers of agriculture, he described his strategy:

We believed that the Permanent Representatives of tobacco growing countries would have strong feelings in defence of their tobacco industry in view of the valuable social and economic benefits which it brings to their countries and that they might discuss the presentation with their ministers [39] (p. 3).

As part of ‘Project Hauser’ [40] McInerney presented his findings in May 1983, comprising one of “two separate and independent studies” [41] (p.2), to “senior members of the FAO and…selected delegates to the FAO from developing countries”.^1^ The presentation stressed the “significant interests to the economy” from “small farmer” employment, export profits, import substitution and tax revenues. The studies were cited as evidence of BAT

playing a positive and substantial role in developing agricultural skills and experience in the developing world. In almost every country in which tobacco is grown and in which BAT has a presence, there is close co-operation and partnership between BAT companies and governments, the farmers and local communities. [42] (p.6)

Dissemination of the studies coincided with the 1983 World Conference on Smoking and Health where industry delegates reported “the anti-smoking movement had come of age” and “many more third world delegates were present” [43]. The conference addressed “the economic theme…in Third World countries”, including a presentation by John Madeley on deforestation in Kenya caused by tobacco farming [44]. It was reported that the paper “created a wave of concern in the international tobacco control community” [45] (p. 191), and elicited a recommendation by delegates that “U.N. agencies must cease supporting tobacco growing and examine deforestation problem” [46].

Despite these growing concerns, however, “a 10-year drought of information on…deforestation caused by tobacco growing and curing in developing countries” ensued, according to *Tobacco Control* editor Simon Chapman [45] (p. 191), during which industry lobbying at FAO continued. The industry pointed to the “lack of research” by tobacco control advocates, and criticised WHO’s “emotional and contradictory…attacks” [47]. Internally, meanwhile, the industry resisted McInerney’s proposal to follow up the desk review with a “properly structured field survey work in key cash crop areas…[with] statistically based results (rather than just anecdotal evidence)” [48]. Ely responded he was “not sure that there would be too much to be gained from it” [49].

Instead, industry efforts were channeled into the development of a “‘pro-active’ EIU follow up strategy” that would “provide the data needed to refute ill-considered WHO policies damaging to the interests of developing countries” [30] (p. 1). Documents suggest a key part of this strategy was the creation by TTCs of the International Tobacco Growers Association (ITGA) in 1984. While the ITGA publicly describes itself as a “non-profit organization…presenting the cause of millions of tobacco farmers to the world” [50], documents show TTCs colluded through the ITGA to “‘front’…our Third World lobby activities at the World Health Organisation, and gain support from nations hostile to multinational corporations” [51]. The ITGA would later play a central role in promoting industry-funded research which downplayed responsibility for deforestation and countered findings from independent research.

### “True causes of deforestation”: downplaying tobacco industry responsibility

Documents suggest that a second tactic of the industry is to attribute deforestation to “many causes” [52] which are far more to blame than tobacco. Recognising the limitations of “denial without documentation” [53], the industry then funded new research, disseminated through front groups, to shape the policy debate. In 1984 Ely [54] commissioned Alistair Fraser, of the International Forest Sciences Consultancy (IFSC), to study the impact of tobacco growing and curing on deforestation. Fraser’s draft report, *The Ecological, Social and Economic Implications of the Use of Wood by the Tobacco Industry* [55], found the impact ranged from none in Argentina to “an important influence” in Malawi. The reported concluded that, “[i]n all the countries studied, the forest resources have been depleted to such an extent that the annual cut of woodfuel appears to exceed the growth in the forest, and forest destruction is taking place, both, through obvious clearance and by the more pernicious overcutting” [55] (p. 47). The study recommended

the tobacco industry assess the true economic cost of the fuel needed for flue-curing the tobacco, taking account of the opportunity cost of the land needed to grow woodfuel, and the foreign exchange implications of using fossil fuels, and develop local long-term strategies to ensure the most economic long-term fuel supply. [55] (p. 47)

Upon submitting this draft, Fraser wrote that “the major part” of his report was “complete,” barring “a short concluding section and bibliography…and possibly some editorial improvements” [56].

However, Fraser’s findings immediately raised concerns among BAT senior staff. Fraser’s “desk research” [57] was considered to contain “data conflicting sharply with” industry sources [58]. Similarly, J. Drummond (BAT Manager of Leaf Department) wrote to Ely:

I am somewhat surprised at the direction Dr. Fraser’s investigations have taken in Thailand….Fraser has made certain suggestions as to future actions. I would be very, very reluctant to support these….we are in a potential mine field and if we were to take up Fraser’s suggestions without a full study of their wider implications….[59].

Despite a FAO report containing data “very close to the ones quoted in my Report”, Fraser agreed to meet with Ely in November 1984 to discuss his estimates of “the quantities of wood-fuel use in flue-curing” [60]. After this meeting, Fraser wrote: “I am very happy to take on board your comments and the additional information which has come to light since submitting the report” [61] (p. 2). The “additional information” proved to be data from local BAT companies [62] “for his consideration” reporting far lower levels of woodfuel use [57].

It was agreed that this new data would be used, funded or provided by the industry, with the methodology approved by BAT [63] including “field work” in case-study countries [64, 65] and “informal approaches to other tobacco curers” [61]. Despite this close industry’s involvement, the study was described by Ely as an “independent survey done on the use of wood fuel for curing tobacco as compared with other fuel and indeed other uses of wood based products” [66] (p. 2).

The revised report, entitled *The use of wood by the tobacco industry and the ecological implications,* revised downward the original finding that “the amount of wood used in flue-curing around the world may be as high as…4.5 % of all woodfuel consumed,” to “less than 1 % of all wood consumption” and “only 0.7 % of all fuelwood consumed” in the case study countries [52]. Fraser was invited to present these findings to an INFOTAB meeting where the evidence was said to show the industry was contributing to deforestation “to a very minor degree” [67] (p. 4). Attendees agreed to submit an article to the *Courier* (published by the European Commission), with copies to FAO and World Bank, based on these findings. Fraser was also to speak to Tanzanian officials as part of Hauser’s lobbying efforts.

The revised report proved especially useful following publication of an 1989 article, “Tobacco: the burning issue” by the UN Department of Information. Douglas Wholey (consultancy firm Minster Agriculture) wrote to Ely describing it as “a very serious affair as the publication is very widely circulated and unfortunately is respected in development circles” [68]. In response, the ITGA published an editorial in its in-house journal, *Tobacco Forum*, which stated:

A lot of nonsense is promulgated about the use of wood by tobacco farmers. Typical of such misinformation, an article published in the UN Department of Information’s ‘Development Forum’…claimed that ‘perhaps one out of every eight trees worldwide is used for curing tobacco’. The fact is that the tobacco industry as a whole accounts for significantly less than 1 % of all wood consumed in the developing world, not all of which is used for curing. The tobacco industry is only one of many industries which use wood as fuel [69] (p. 1).

Fraser also wrote to the *Development Forum* editor challenging the article’s claims:

In 1986 we were invited to carry out a totally independant [sic] survey of the use of wood by the tobacco industry….the tobacco industry accounts for rather less than 1 % of all wood consumed in the developing world, rather than the 12 % implied by the one tree in eight in your article. It does harm to the credibility of the U.N. system when such articles are published which do not indicate the sources of the information, and which are not based on the scientific data which is available [70] (p. 1–2).

Sending a copy of Fraser’s letter to Wholey, Ely wrote: “hope you will agree with me that it is a very complete answer. However, in case it is not published we will have to take fresh steps to cope with the situation” [71]. This included the ITGA inviting leaf growers to “place *Tobacco Forum* in appropriate hands within your country. For example, with local newspapers/magazines, opinion-formers, relevant government officials” [72]. This was followed in 1990 by the ITGA publishing an atlas on leaf growing, *Tobacco in the Developing World*, “to convince First World governments and UN agencies that their anti-tobacco campaigns are bad news for the developing countries that grow tobacco” [73]. The atlas was updated and re-issued several times over the next decade. TTCs were, in turn, advised to use ITGA publications to downplay industry responsibility. For BAT, for example, the advised model response was:

The tobacco industry does not use rain forests to cure tobacco and does not grow tobacco in rain forest areas because the climate is not suitable. In other areas it is BAT policy not to clear forestry land. Only land already cleared and being used for agriculture is considered for use [74] (p. 1).

Over the next decade, frequent referral to Fraser’s “independent study” [75] was made to dispel “woodfuel myths” [76] (p. 3), using “objective third party data” [77], and to draw proper attention to the “true causes of deforestation” [78] (p. 28), namely “population growth, non-sustainable agricultural practices and lack of affordable alternative fuels” [79].

By the early 1990s, non-industry funded research began to report alarming rates of deforestation in Kenya, Tanzania and Uganda. Funded by the Panos Institute, launched at the 1993 All Africa Conference on Tobacco and Health, and published in *Tobacco Control*, a case study of two regions in Tanzania found tobacco “had turned the area into an environmental disaster” [80] (p. 252). In Uganda’s West Nile region, which produces 80 % of the country’s tobacco, natural forest cover was reduced from 7225 to 3000 ha. Up to 130 kg of woodfuel was being used to cure 1 kg of tobacco [81]. In Kenya, indigenous trees were “severely reduced in numbers” from expanded tobacco growing [82] (p. 249).

These renewed concerns were initially dismissed as opportunism by public health advocates “seeking to take advantage of” the 1992 UN Conference on Environment and Development (UNCED) [83] (p. 3). As new evidence emerged, however, the industry took a more strategic approach. The consultancy Agro-Tobacco Services (ATS) was formed in 1992, with £426,000 from six tobacco companies (BAT, PM, RJ Reynolds, Rothmans International, Gallaher and Reemtsma), along with “international leaf dealers, state-controlled tobacco manufacturers, and other tobacco interests” [84] (p. 12). Headed by ITGA Assistant Secretary General Martin Oldman, ATS’s role was to “develop and implement action plans for each of the ITGA member organizations, develop new argumentation, and liaise with external allies” [85]. Ahead of the 1993 All Africa Conference, a briefing paper was developed “for placement before and/or during the Conference”, aimed at “rebalancing some misconceptions regarding tobacco and wood use” [86] (p. 1). To counter publication of *Tobacco: The Smoke blows South* by the Panos Institute [87], updating the report on Uganda [88], Oldman published *Developing Countries and Tobacco* as “a critical analysis” which “ITGA/Agro-Tobacco Services could usefully deploy 500 copies” [89] (p.1). The report described the principal author of the Panos report, John Madeley, as “a well known anti-tobacco lobbyist [who] makes a wide range of very predictable claims against the tobacco industry.” In contrast, despite being a longstanding ITGA employee, Oldman is described as an “independent consultant specialising in tobacco-attributable issues in the developing world” using “independently published sources” [89] (p. 4).

### Forestation as “an important PR tool for marketing the Corporate Brand”

A third key tactic of the tobacco industry, to counter deforestation concerns, was involvement in, and even leadership of, forestation efforts (reforestation and afforestation). The industry rejected the “rash of stories condemning transnational tobacco company activities in developing nations” [90] (p. 2), instead blaming lack of government action for deforestation:

Where Third World governments have generally encouraged the development of tobacco, their forestry departments have often been slow to recognize the need for reforestation. Tobacco companies have, therefore, taken the initiative, encouraging farmers to plant trees either individually or on a cooperative basis, even providing free seedlings for both depleted forestland and new land [90] (p. 12–13).

This portrayal of the industry as responsible corporate citizen was supported by the initiation of forestation projects in several countries including Nigeria [91], the Philippines [92], and Sri Lanka [93]. In the Dominican Republic, PM’s “Institutional Reforestation Campaign” was featured on television, newspaper advertisements, posters and t-shirts [94] (p. 38). In Brazil, BAT subsidiary Souza Cruz claimed to have replanted by 1984 “much more than the total amount of wood needed for every crop….forests have been increasing instead of diminishing” [95] (p. 2). The claimed success of BAT’s program in Kenya led the Tanzanian Permanent Representative to the UN in Geneva to request “human and financial resources…for an afforestation program” towards “arresting or minimising the environmental degradation that tobacco growing causes in developing countries” [96] (p. 1).

Over the next decade, there was little scrutiny and no independent research to counter industry forestation claims. One exception was a 1984 report by Madeley which argued that BAT’s reforestation plans in Kenya were “going very badly”. He reported that land growing tobacco was five times more than for trees. Around 12 million trees were cut annually for curing, causing desertification, displacement of food crops and woodfuel shortages [44] (p.2). Into this vacuum BAT “began to make use of” a commissioned study [97], again presented as an “independent audit”, by Moi University “to demonstrate BAT Kenya’s contribution” [98] (p. 2). It was not until the mid 1990s that hard questions about the effectiveness of industry-led forestation projects were raised. For example, Kweyuh observed that afforestation “largely failed to achieve the desired effect” [82] (p. 249). In Tanzania, Waluye observed that “very few trees are planted annually compared to those cut down” [80] (p.253). In 1993, the Panos Institute reported:

In Kenya…BAT says that farmers can only become tobacco farmers if they agree to plant 1000 eucalyptus trees a year on their land. But there are problems. The average smallholder in Kenya has less than 4 ha of land. If he or she plants tobacco, that might take up half a hectare and the trees a further hectare. Land for food and other purposes is squeezed….[A] former senior employee of BAT Kenya alleged: “the company is shouting about massive tree planting but this I’m afraid is nothing less than an outrageous attempt to veil the whole problem” [87] (p. 11).

To counter these criticisms, the ITGA showcased “18 countries running afforestation programmes PR [public relations]” for BAT [99] (p. 1). BAT claimed it “helped to plant more trees than are used to cure the tobacco it buys….over 100 million surviving trees…forecast to increase to 152 million trees by 1995” [100] (p. 2). Moreover, “no farmer will be recruited to grow tobacco for BAT unless he/she has satisfied the minimum requirements for trees” [101]. The programmes would “provide enough woodfuel for…contracted farmers to be 26 % self sufficient” [100] (p. 2), rising to 53 % by 1995 [99] (p.1) and 81.9 % by 2000 [102] (p. 25). In this way, it was expected that BAT could use the claim of “close to a ‘carbon balance’ in its operations” as “an important PR tool for marketing the Corporate Brand” [102] (p. 4). Documents describe BAT making afforestation one of its “global flagship CSR programmes” in 1999 as “an area of frequent attacks against us by pressure groups” [103] (p. 4). An Afforestation Team was formed, working closely with the EarthWatch Institute as “a member of the project team” [103] (p. 5). In 1999 BAT hosted Afforestation Week in Uganda [104], and was keen to “exploit our good efforts” by sending “the head of ‘Earthwatch’ to take a look” [105] (p. 1). Following a similar visit to Ghana, EarthWatch African Programme Manager Lucy Beresford-Stooke wrote, “BAT appear to be actively facing environmental issues” [106] (p.2).

Internally, however, BAT staff expressed concerns. While the ITGA publicly stated that “the wood deficit [in Zimbabwe] is not so serious and alternative fuels are available” [107], Parirewa of BAT Zimbabwe admitted,

Sadly, the survival rate of the trees is almost zero as attention given to the woodlots is poor. Nonetheless, British American Tobacco Zimbabwe must be seen to be concerned about the depletion of forests although it has no direct link with tobacco growers who are considered the main “culprits’ in the cutting down of trees [108] (p.1).

In Pakistan and Nigeria, it was acknowledged that efforts “will fall well short of the afforestation policy target of self-sufficiency in wood-fuel usage by 2000”, and there was “need to introduce urgent corrective action” [102] (p. 4). In 2001 preservation of natural forests became part of its “Sustainable agriculture and farmer livelihoods” initiative and Biodiversity Partnership with the Earthwatch Institute, Fauna & Flora International, the Tropical Biology Association [109]. Since the late 1990s, non industry-funded studies confirmed the alarming situation [110]. A 1999 study by University of Düsseldorf geographer Helmut Geist found the industry caused 4.6 % of deforestation in developing countries and 12 % in southern Africa [8, 111]. Geist’s findings were widely cited including in the report *Golden Leaf, Barren Harvest*:

[D]espite government recommendations to have 10 % of farm land planted with trees, studies by the Extension Service of Malawi found that 80 % of estate farmers had failed to follow this advice. In Tanzania’s Southern Songea highlands, where most tobacco is fire-cured, Geist found that a mere 1.4 % of the overall farmland of tobacco growers was planted with trees, as opposed to the officially mandated 20 % [112] (p. 26).

Samson Mwita Marwa, a Kenyan tobacco farmer and former Member of Parliament, observed: “BAT claims to be engaged in reforestation programmes. I have yet to see a single mature tree that BAT has planted in Kuria district….the rate of deforestation is far too fast to be equal to the rate of reforestation” [113] (p. 2). Bates et al. reported that

In Uganda, BAT has been planting the fast growing eucalyptus trees to replace depleted indigenous species like the shea butter tree whose oil is used in cooking in many parts of Northern Uganda. The eucalyptus is an anti-social thirsty tree. Its fast growth rate places a great demand on the soil water and nutrients, while its fallen leaves contain chemicals that discourage the growth of other vegetation near the tree [114] (p. 3).

Sauer and Abdallah found 69 % of tobacco farmers in the Urambo District, Tanzania clear new woodlands for cultivation every season, because virgin land offers fewer soil-borne diseases and higher yields, with 25 % using the same plot for two consecutive seasons [115]. WHO concluded that “[r]eforestation programmes cosponsored and promoted by the tobacco industry are not enough to reverse the damage” [113] (p. 2). Kweyuh argued that the “Industry should be forced to withdraw all phony statistics and research and to finance state and NGO initiatives to address the deforestation” [116].

With this accumulating evidence, TTCs began to publicly acknowledged the severity of the problem. A 2012 BAT-commissioned review concluded that deforestation “may be the single most negative impact of tobacco cultivation on the environment” [117] (p. 6). The report found that the “‘continuous reduction’ of wood use in curing green leaf tobacco, as claimed in industry commissioned works such as those of Fraser [52] and Campbell [75], can very likely not be substantiated given the results of the study by Geist et al. [111]” [117] (p. 12). Today BAT admits that “[l]oss of natural forests is one of the most significant environmental impacts linked to tobacco growing, due to wood often being used as a fuel in curing processes” [118]. Similarly PM reports that “tobacco farming has contributed to extensive depletion of [Malawi’s]…trees due to the use of large quantities of wood in traditional tobacco curing process” [119]. Japan Tobacco International (JTI) recognises:

[I]n Malawi, forest cover has more than halved over the last four decades, creating fears about the sustainability of wood as a resource. This has significant consequences for the lifestyles and livelihoods of the farmers JTI works with. Without a ready supply of wood they are less able to sustain their way of living and produce the high volume of quality tobacco that both they and JTI need [120].

Alliance One International, a leaf merchant in more than 45 countries, notes: “The lush forests that once spread across Kenya’s landscape are now vanishing at an alarming rate. While environmental impacts of deforestation border upon dismal, the lack of trees also threatens the entire country with drought” [121].

## Discussion

To date, analyses of efforts by the tobacco industry to influence research and policy have been largely focused on public health issues. We demonstrate in this paper that tobacco industry efforts to influence public policy extend to the environment sector.

The above findings suggest that the tobacco industry sought to shape understanding of the deforestation problem, caused by tobacco farming and curing, from the 1970s to early 2000s centred on claims of economic benefits [122], alternative causes of tobacco-related diseases [123]; and responsible behaviour towards consumers and communities [124]. These arguments were advanced through lobbying at the national and international levels, collusion through the use of front groups [125], and industry commissioned research by paid consultants [16, 126]. These are all well-documented tactics, used by the industry to prevent or delay regulation on other tobacco control issues, although to date analysed to a limited extent in relation to environmental issues.

The effectiveness of efforts to downplay tobacco-attributable deforestation, against claims of economic benefits to farmers and LMIC governments, was enabled by the latter’s contractual dependence on global markets controlled by large leaf processing and manufacturing oligopolies. The blame placed on local communities and national governments shifted attention away from their subsistence-level dependence, “steered overproduction”, and lack of alternative fuel sources and livelihoods. The claims that industry-led forestation initiatives effectively addressed the problem left primary responsibility to smallholder farmers incapable of growing sufficient trees to compensate for the scale of deforestation. This shaping of the deforestation issue was underpinned by commissioned research whose findings were altered to suit industry interests. From the 1970s onwards, this “independent research” was disseminated to senior UN and government officials by front groups and paid consultants. The overall aim, as described by PM, was “to manage and mitigate punitive regulatory measures, enhance the corporate image, [and] influence public policy” [127].

The lack of effective action to address tobacco-attributable deforestation, and indeed prevent escalation of the problem [8, 115], can be understood in this context. Along with insufficient national responses, international policies encouraged the expansion of global leaf production. World Bank financing supported tobacco growing until 1991. Trade agreements, such as the Cotonou Agreement of 2000 between the European Union and LMICs, created favourable terms for tobacco exports. As Otanez et al. explain,

The trade policies that reflect the interests of developed countries and corporations in the current global economic system contribute to poverty in Malawi and other developing countries because these countries produce commodities for export markets instead of food for their own consumption, and depend on costly agricultural chemicals that pollute water supplies, erode soils and contribute to deforestation [25] (p. 262).

These findings have important implications for understanding the role of the tobacco industry in environmental policy. First, there is a need to more fully measure the diverse environmental consequences of the tobacco industry over time. Tobacco-attributable deforestation warrants the urgent collection of systematic data across time and place by independent scientists. Other environmental impacts include agrochemical pollution [128], loss of biodiversity [129], air pollution [130] and waste toxicity [131]. The limited and fragmented nature of available data since the 1980s enables the industry to continue to downplay its environmental impacts and offer industry-serving solutions.

Second, the above findings suggest that the involvement of some environmental scientists and groups played an important role in the industry’s tactics, by lending support to perceptions that industry-commissioned research and policy initiatives are scientifically-based and credible. As described by McDaniel and Malone, it is important that environmental groups be aware of how they provide legitimacy and help redeem the industry’s negative public reputation [132]. These links with environmental groups can also provide an entrée for the tobacco industry into civil society and CSR, thus avoiding direct responsibility for the environmental consequences of the industry.

Third, environmental policy research has understandably given growing attention to the political power of the business sector [133], focusing on industries such as oil and gas [134] and agrochemicals [135]. Scrutiny of the tobacco industry has traditionally come from the public health community. These findings demonstrate the need for the environmental community to include the tobacco industry in efforts to improve the transparency and accountability of corporations.

## Conclusions

Deforestation remains a serious global environmental challenge. Available evidence suggests tobacco farming and curing, to produce a product that kills 6 million people annually, accounts for 4 % of deforestation globally, and 18–30 % in some LMICs. There have been limited efforts to date to address tobacco-attributable deforestation as a problem, led by CSR initiatives by the industry. The findings of this paper suggest that this is due, in part, to the tobacco industry shaping understanding of the deforestation problem, and potential solutions to address it, in ways that sustain the interests of the industry. There is an urgent need for independent measurement and monitoring of the problem, particularly in high production LMICs, and a range of policy measures which address the dependency of tobacco farmers on powerful global leaf processors and manufacturers.

## Abbreviations

ATS: Agro-Tobacco Services
BAT: British American Tobacco
CSR: Corporate social responsibility
EIU: Economic Intelligence Unit
FAO: Food and Agriculture Organization
GDP: Gross domestic product
ICOSI: International Committee on Tobacco Issues
IFSC: International Forest Sciences Consultancy
IMF: International Monetary Fund
INFOTAB: International Tobacco Information Centre
ITGA: International Tobacco Growers Association
JTI: Japan Tobacco International
LMIC: Low and middle income country
PMI: Philip Morris International
TAC: Tobacco Advisory Council
TTC: Transnational tobacco company
UNCED: UN Conference on Environment and Development
WHO: World Health Organization

## Footnotes

1 For a list of participants see BAT (1984). Participants at Mr. Hauser’s Reception.” http://legacy.library.ucsf.edu/tid/cfc44a99/pdf
